# Cdk5 phosphorylation‐dependent C9orf72 degradation promotes neuronal death in Parkinson's disease models

**DOI:** 10.1111/cns.14319

**Published:** 2023-06-23

**Authors:** Xingfeng Xu, Mao Li, Yan Su, Qi Wang, Peifang Qin, Haitao Huang, Yuting Zhang, Yali Zhou, Jianguo Yan

**Affiliations:** ^1^ Department of Physiology Guilin Medical University Guilin Guangxi China; ^2^ Guangxi Key Laboratory of Brain and Cognitive Neuroscience Guilin Medical University Guilin Guangxi China; ^3^ Department of Microbiology Guilin Medical University Guilin Guangxi China

**Keywords:** C9orf72, cyclin‐dependent protein kinase 5, Parkinson's disease, protein phosphorylation, ubiquitin‐proteasome pathway

## Abstract

**Aims:**

Chromosome 9 open reading frame 72 (C9orf72) is one of the most dazzling molecules in neurodegenerative diseases, albeit that its role in Parkinson's disease (PD) remains unknown. This article aimed to explore the potential mechanism of C9orf72 involved in the pathogenesis of PD.

**Methods:**

The expression and phosphorylation levels of C9orf72 were examined by Western blotting, RT‐PCR, and immunoprecipitation using PD models. Multiple bioinformatics software was used to predict the potential phosphorylation sites of C9orf72 by Cdk5, followed by verification of whether Cdk5‐inhibitor ROSCOVITINE could reverse the degradation of C9orf72 in PD. By constructing the sh‐C9orf72‐knockdown adenovirus and overexpressing the FLAG‐C9orf72 plasmid, the effects of C9orf72 knockdown and overexpression, respectively, were determined. A short peptide termed Myr‐C9orf72 was used to verify whether interfering with Cdk5 phosphorylation at the Ser9 site of the C9orf72 protein could alleviate autophagy disorder, neuronal death, and movement disorder in PD models.

**Results:**

The expression level of the C9orf72 protein was significantly reduced, albeit the mRNA expression was not changed in the PD models. Moreover, the phosphorylation level was enhanced, and its reduction was mainly degraded by the ubiquitin‐proteasome pathway. The key nervous system kinase Cdk5 directly phosphorylated the S9 site of the C9orf72 protein, which promoted the degradation of the C9orf72 protein. The knockdown of C9orf72 aggravated autophagy dysfunction and increased neuronal loss and motor dysfunction in substantia nigra neurons of PD mice. The overexpression of C9orf72 alleviated autophagy dysfunction in PD neurons. Specifically, interference with Cdk5 phosphorylation at the S9 site of C9orf72 alleviated autophagy dysfunction, neuronal death, and motor dysfunction mediated by C9orf72 protein degradation during PD.

**Conclusions:**

Cumulatively, our findings illustrate the importance of the role of C9orf72 in the regulation of neuronal death during PD progression via the Cdk5‐dependent degradation.

## Graphical abstract

The theoretical scheme of Chromosome 9 open reading frame 72 (C9orf72) protein involved in neuronal death in Parkinson's disease (PD). During PD, Cdk5 is activated in dopaminergic neurons of the substantia nigra, and the activated protein kinase Cdk5 phosphorylates the S9 site of C9orf72 protein. Phosphorylation at S9 site mediates C9orf72 protein degradation through the ubiquitin‐proteasome pathway. Degradation and loss of function of C9orf72 protein, leading to autophagy impairment in neurons. Proteins such as p62 and α‐synuclein that cannot be effectively cleared by autophagy accumulate abnormally, and their neurotoxicity causes the death of dopaminergic neurons.

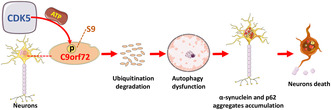

## 1 INTRODUCTION

Parkinson's disease (PD) is a common progressive neurodegenerative disease with an insidious onset. PD more commonly affects the elderly, with an average age of onset of 60 years.^1^ PD, also known as tremor palsy, is clinically characterized by resting tremors, bradykinesia, rigidity, and postural gait disturbance. The most important pathological changes observed in PD are the degeneration and death of dopaminergic neurons in the substantia nigra, which, subsequently, markedly reduces the striatal dopamine content and the formation of Lewy bodies in neurons.^2^, ^3^, ^4^ Although several past studies have been conducted on PD, the specific mechanisms underlying dopaminergic neuron death are yet to be studied.

According to recent studies, C9orf72 is closely related to the pathogenesis of neurodegenerative diseases.^5^, ^6^, ^7^ The C9orf72 protein expression is reduced in patients with amyotrophic lateral sclerosis (ALS) and frontotemporal dementia (FTD),^8^, ^9^, ^10^ while the knockdown of C9orf72 causes the dysfunction of endocytosis and autophagy.^11^ Neurons in FTD/ALS patients exhibit impaired basal autophagy^12^ and increased sensitivity to autophagy inhibition,^13^ implying that reduced levels of C9orf72 can cause neuronal cell damage.

Past studies have demonstrated that Rab proteins 1, 5, 7, and 11 regulate autophagy^14^, ^15^, ^16^ in the mouse glioma cell line Neuro2A, human glioma cell line SH‐SY5Y, primary cortical neurons, and spinal cord motor neurons, which colocalizes and directly interacts with C9orf72. Other scholars have reported that C9orf72 can induce the activation and translocation of ULK by directly interacting with the ULK complex.^11^, ^17^, ^18^ Importantly, C9orf72 mainly plays an early role in autophagy at the stage of autophagosome formation, and the knockdown of C9orf72 in human cell lines and primary neurons specifically inhibits autophagy induction, which, in turn, promotes the accumulation of p62.^11^, ^19^ Consistent with these findings, the accumulation of autophagy substrates, such as p62, has been recorded in C9orf72‐knockout mice.^20^ The decreased expression of C9orf72 has been reported to specifically inhibit autophagy, promote the accumulation of dipeptide repeat protein aggregates (DPR), and cooperate with DPR to produce low toxicity of DPR protein.^21^ Conversely, the overexpression of C9orf72 can activate autophagy, subsequently increasing the autophagosomes in cell lines.^19^

In this study, we noted that the C9orf72 protein expression was significantly reduced in various PD animal and cellular models, accompanied by autophagy dysfunction and the accumulation of autophagy substrates. Further research revealed that the downregulation of C9orf72 expression led to autophagy dysfunction, while the overexpression of C9orf72 led to the activation of autophagy and the promotion of the degradation of autophagic substrates. Numerous studies have suggested that, during PD, Cdk5 is overactivated in the brain and its phosphorylation function is enhanced.^22^, ^23^, ^24^ In this experiment, we determined that Cdk5 activated during PD phosphorylated the S9 site of C9orf72. Taken together, our results suggested that autophagy impairment induced by the degradation of C9orf72 during PD is dependent on the regulation of Cdk5 activity.

## 2 MATERIALS AND METHODS

### 2.1 Antibodies and chemical reagents

The antibody of β‐actin (WB‐1: 5000, A5441), MPTP, and MPP^+^(Lot#134347) were obtained from Sigma‐Aldrich. The antibodies of rabbit polyclonal anti‐p62 (WB‐1: 1000, 23214S) and LC3 (WB‐1: 1000, 3868S) were purchased from Cell Signaling. Rabbit polyclonal anti‐α‐synuclein (WB‐1: 800) was obtained from Proteintech. Rabbit polyclonal anti‐C9orf72 (WB‐1: 800, A15970) was purchased from Abclonal. Mouse anti‐tyrosine hydroxylase (WB‐1: 5000) was obtained from Millipore. The antibodies were used at the dilutions recommended by the manufacturers. All WB secondary antibodies (anti‐mouse‐1:5000, anti‐rabbit‐1:10000) were procured from Jackson ImmunoResearch. All fluorescent secondary antibodies (1:500) were procured from Jackson ImmunoResearch and used according to the manufacturer's instructions. MG132 was purchased from Abcam. 3‐Methyladenine (3‐MA) was obtained from Calbiochem. ROSCOVITINE was procured from Sigma. The antibodies against GFP and Flag tag were purchased from Proteintech.

### 2.2 Plasmids, virus, and recombinant proteins

The plasmids encoding GFP‐C9orf72, Flag‐C9orf72 WT, and Flag‐C9orf72 S9A were purchased from Brain Case. The plasmids encoding GFP‐vector, GFP‐α‐synuclein A53T, and HA‐Cdk5/Myc‐p35 were owned by our research group. The EGFP‐α‐synuclein‐A53T adenovirus was purchased from BrainVTA. The Sh‐C9orf72 adenovirus (AAV2/9) was purchased from Brain Case. Myr‐C9orf72 protein undecapeptide (amino acids 4–14, sequence LCPPPSPAVAK, P210824‐LL926654) and Scramble‐C9orf72 protein undecapeptide (P210824‐LL926655) were purchased from GL Biochem (Shanghai) Ltd.

### 2.3 Animals, MPTP injections, and stereotactic injection

Male C57BL/6 mice (age: 8 weeks: weight: 22–28 g) were used to mimic an acute PD mouse model (mice purchased from Hunan Sja Laboratory Animal Co., Ltd. SCXK (Xiang) 2019–0004). The mice received a single intraperitoneal injection of 25 mg/kg MPTP once a day for 10 consecutive days, and 0.9% saline was used as the vehicle. MPTP was administered to 3‐month‐old male C57BL/6 mice (weight: 22–28 g) to mimic a chronic PD mouse model.^22^ These mice received an intraperitoneal injection of 250 mg/kg probenecid 10 times over 5 weeks, with 0.03 mL DMSO used as the vehicle 30 min after probenecid injection, followed by the subcutaneous injection of 25 mg/kg MPTP. Stereotaxic injection of adenovirus α‐synuclein A53T (the injection coordinates were the SNpc brain area, AP: 3.16 mm, ML: 1.125 mm, DV: 4.43 mm) was used to establish a genetic model of PD. Stereotactic injection of recombinant α‐synuclein PFF (ab246002, a‐syn preformed fibrils, the injection coordinates were the SNpc (substantia nigra pars compacta) brain area, AP: 3.28 mm, ML: 1.35 mm, DV: 4.5 mm) was used to establish a preclinical PD model. Stereotaxic injection of Sh‐C9orf72 adenovirus (the injection coordinates were the SNpc brain area, AP: 3.16 mm, ML: 1.125 mm, DV: 4.43 mm) was used to establish a C9orf72 knockdown PD animal model.^22^

### 2.4 Cell culture and transfection

The neurons were cultured at embryonic days 16–18 from Sprague–Dawley rats on 6‐well plates precoated with poly‐D‐lysine (Sigma‐Aldrich). The primary neurons were maintained in the neurobasal medium (Gibco) containing 2% B27 supplement (Shanghai Biogene Biotech), 0.5 mM glutamine (Gibco), and 25 μM penicillin and streptomycin (100 g/mL; Solarbio). After 24 h of plating, the cell division inhibitor Ara‐C was added to the medium at a final concentration of 10 μM to remove the glial cells. All treatments or transfections were performed after 7 days of in vitro plating. HEK293 cells were cultured in DMEM(Lot#8122280) supplemented with 10% FBS and the SH‐SY5Y cells were cultured in DMEM/F12 supplemented with 10% FBS. HEK293, SH‐SY5Y cells and primary neurons were transfected with the indicated plasmids with the Lipofectamine 2000 reagent (Sigma) in accordance with the manufacturer's instructions.

### 2.5 Immunofluorescence staining

For tissue staining, the mice were anesthetized with 5% chloral hydrate and perfused first with normal saline and then with 4% PFA for 8 min. The brain tissues were carefully removed, placed in 4% PFA, fixed at 4°C for 24 h,^22^ and treated with 20% sucrose solution to dehydrate the brain tissues. The dehydrated rat brain was embedded and fixed on an ice holder, and the substantia nigra was removed from the substantia nigra on a cryostat for continuous coronal sections of 15 μm (Leica CM1850), attached to a glass slide, and then dried in a 55°C oven. For cell staining, the cells were cultured on glass coverslips, and, after the appropriate treatment, the cells were fixed on coverslips with 4% PFA. The slides were then treated with 1% SDS for antigen retrieval for 5 min and then washed thrice with PBS, 5 min each time. The slide was then incubated with 0.5% TritonX‐100 (P0096) for 15 min to rupture the membrane and blocked with 10% goat serum (plus 0.5% TritonX‐100) for 1 h at room temperature. Then, 10% goat serum was used to prepare the primary antibody working solution (the dilution concentration of each antibody was mouse anti‐TH 1:250, rabbit anti‐C9orf72 1:100) and incubated overnight at 4°C. After overnight incubation with the primary antibodies, 21 Alexa Fluor® 594‐labeled goat anti‐mouse IgG (H + L) and 21 Alexa Fluor® 488‐labeled goat anti‐rabbit IgG (H + L) (Jackson ImmunoResearch Inc.) were incubated with fluorescent secondary antibodies for 1 h at room temperature in the dark (the dilution ratio of the fluorescent secondary antibody was 1:500, and PBS was used as the antibody diluent to prepare the fluorescent secondary antibody working solution). Then, DAPI (Solarbio, S2110, 10 μg/mL) was added dropwise to the brain slices for nuclei staining for 10 min, to which 1–2 drops of anti‐fluorescence quencher were added to seal the slides. After the filming, the samples were collected and observed under the Olympus upright fluorescence microscope, followed by image processing with Image J software.

### 2.6 In vivo ubiquitination assay

1 μM 3‐MA was added to the MPP^+^ or A53T‐treated neurons for 7 days; 20 μM MG132 was added to the MPP^+^ or A53T‐treated neurons for 7 days. Then, the neurons were lysed in the RIPA buffer, and the lysate was boiled in the SDS sample buffer for 5 min, followed by being subjected to Western blotting with the corresponding antibodies.

### 2.7 Immunoprecipitation, co‐immunoprecipitation, and Western blotting

The midbrain substantia nigra proteins and neuronal cortical cells were cleaved in RIPA buffer (Solarbio, R0010) containing protease inhibitors. The lysates were incubated with the corresponding antibodies overnight at 4°C and then spun with protein G plus/protein A‐agarose (Calbiochem) for 3 h at 4°C. After washing thrice, the immune complexes were boiled in an SDS sample buffer for 10 min, subjected to SDS‐PAGE, transferred onto a nitrocellulose membrane, and then immunoblotted with the corresponding antibody. The primary cortical neurons were co‐transfected with the Flag‐C9orf72 WT plasmid and Flag‐C9orf72 S9A plasmid, while the HEK293 cells were co‐transfected with HA‐Cdk5/Myc‐p35 plasmid. Next, the cells were treated with MG132 (20 μM) for 24 h. The primary cortical neurons and HEK293 cells were lysed in RIPA buffer (a portion of the lysate was reserved for WB), and immunoprecipitation was performed with the corresponding antibody and protein G plus/protein A‐agarose. The immune complexes were boiled in the SDS sample buffer for 10 min, subjected to SDS‐PAGE, transferred onto a nitrocellulose membrane, and finally immunoblotted with the corresponding antibody. The remaining lysate was boiled in the SDS sample buffer for 5 min and subjected to Western blotting with the corresponding antibodies.

### 2.8 Quantitative real‐time PCR analysis

The total RNA was extracted from SNpc with the TRIzol reagent (Invitrogen). The total RNA (1 μg) of each sample was reverse‐transcribed using the MonScript™ RTIII 5× All‐in‐One Mix (Monad Biotech Co., Ltd.) in a 20‐μL volume. For PCR, the amplification was performed in a total volume of 20 μL containing 0.4 μL of each primer,^25^ X μL of cDNA, 10 μL of the MonScriptTM SYBR Green qPCR Mix, and Nuclease‐Free Water to a 20‐μL volume, as per the manufacturer's instructions. GAPDH was amplified as a reference standard for the mice. The primers used in the study were as follows: for rat GAPDH forward: 5’‐TGAAGGGTGGGGCCAAAAGG‐3′, reverse 5′‐ GGTCATGAGCCCTTCCATGA‐3′; for mice GAPDH forward: forward 5’‐GGTTGTCTCCTGCGACTTCA‐3′, reverse 5′‐ TGGTCCAGGGTTTCTTACTCC‐3′; for human GAPDH forward: forward 5’‐GGGTGGGGCTCATTTGCAGGG‐3′, reverse 5′‐ TGGGGGCATCAGCAGAGGGG‐3′; for rat C9orf72 forward: forward 5’‐GTGTTGACAGGCTAACGCAC‐3′, reverse 5′‐ AGGGATGACCTCCCCAGTAA‐3′; for mice C9orf72 forward: forward 5’‐CCTGATGTCAGGTGCATCGT‐3′, reverse 5′‐ GAGGGGCAGGAAGTCAACTC‐3′; for human C9orf72 forward: forward 5’‐GTTGATAGATTAACACATATAATCCGG‐3′, reverse 5′‐ CAGTAAGCATTGGAATAATACTCTGA‐3′. The PCR cycling conditions were 40 cycles of pre‐denaturation at 95°C for 30 s, 90°C for 10 s, 60°C for 10 s, and 72°C for 30 s.

### 2.9 Cell survival assay

The experimental cells were grouped as follows: Vector, FLAG‐Vector plasmid+MPP^+^, FLAG‐C9orf72 plasmid+MPP^+^, FLAG‐Vector plasmid+PFF, FLAG‐C9orf72 plasmid+PFF, and FLAG‐C9orf72 plasmid. When the cells grew to 70%–80% confluence of the slide, the cell solution was changed to MEM medium without serum and double antibodies, and the transfection was performed after 2 h. Then, 1.5‐mL sterile EP tubes were labeled “1” and “2”, and 250 μL of the Opti‐MEM was added to each tube, 5–10 μL of Lipofectamine2000 was added to “tube 1” and 150 pM of FLAG‐C9orf72 plasmid to “Tube 2”. The two tubes were left to stand for 5 min, then both tubes 1 and 2 were mixed thoroughly, and allowed to stand for 20 min. The cells were then washed in a 6‐well plate twice with PBS, followed by the addition of 1.5 mL of serum‐free medium. Then, the mixture of tubes “1” and “2” was added to the 6‐well plate gently and uniformly with continuous culture for 6 h. After 6 h, the serum‐free medium was changed to a normal medium (without the addition of a double antibody) and the culture was continued for 18 h. After 24 h of transfection with FLAG‐Vector plasmid and FLAG‐C9orf72 plasmid, the 6‐well plate was removed and 1 mM of MPP^+^ or PFF was added to each well for 24 h. Each group was designed with 3 parallels. After 24 h, the culture medium was discarded, and the fresh complete culture medium was mixed with CCK‐8 at 100 μL: 10 μL. Next, 110 μL of the mixture was added to each well, and the culture was continued for 4 h. The OD value of each well was measured with a microplate reader at the wavelength of 450 nm. Cell viability (%) = (experimental Group A–blank group A)/(control group A–blank group A) × 100%.

### 2.10 Open field experiment

The mice were placed in an open field (50 × 50 × 25 cm) and free to move for 15 min, a video camera was used to record the movement path and total distance of the mice. Keep the environment quiet during the experiment, so as not to frighten the mice and affect the experimental results, and repeat the test every 2 days. At the end of each mouse's test, the feces and urine of the mouse were cleaned, and the field was sprayed with 75% alcohol to eliminate the smell of the previous mouse.

### 2.11 Rotarod test

Mice were placed on the roller in turn, and then the speed of the roller was gradually adjusted to 30 rmp/min at an acceleration of 0.3 rmp/sec, and the time from the start of the roller rolling to the drop of the roller was recorded. Mice in each group were tested for 5 times, with the maximum value of 5 min each time, and the average value was taken as the final test result. The mice would crawl around on the roller, and as the speed of the roller increased, the mice eventually lost their balance and fell off the roller, and stop timing at this point. The apparatus was cleaned with ethanol between each test.

## 3 RESULTS

### 3.1 C9orf72 protein was decreased in animal models of PD

**FIGURE 1:**
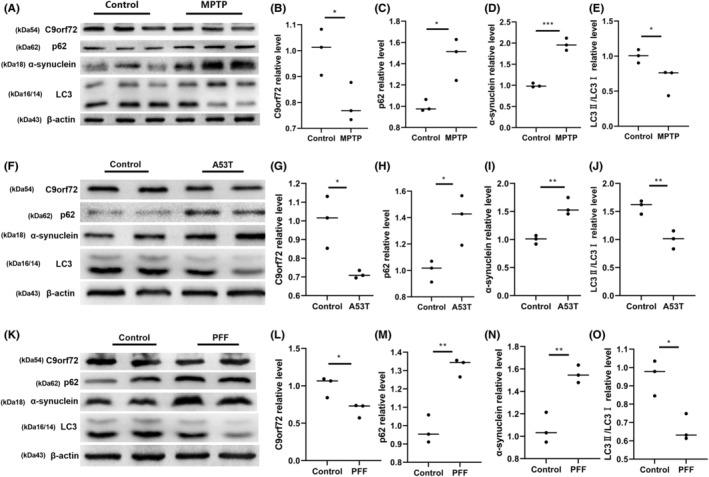
Chromosome 9 open reading frame 72 (C9orf72) expression is decreased in Parkinson's disease (PD) animal models. (A) Western blotting of C9orf72, p62, α‐synuclein, and LC3 in MPTP‐induced PD mice. (B) Statistical analyses of the relative content of C9orf72 in MPTP‐induced PD mice. (C) Statistical analysis of the relative content of p62 in MPTP‐induced PD mice. (D) Statistical analysis of the relative content of α‐synuclein in MPTP‐induced PD mice. (E) Statistical analysis of the relative content of LC3 in MPTP‐induced PD mice, *n* = 3/group. *p < 0.05. (F) Western blotting of C9orf72, p62, α‐synuclein, and LC3 in α‐synuclein A53T‐induced genetic PD mice. (G) Statistical analysis of the relative content of α‐synuclein A53T‐induced C9orf72. (H) Statistical analysis of the relative content of α‐synuclein A53T‐induced p62. (I) Statistical analysis of the relative content of α‐synuclein A53T‐induced α‐synuclein. (J) Statistical analysis of the relative content of α‐synuclein A53T‐induced LC3, *n* = 3/group, **p < 0.01, *p < 0.05. (K) Western blotting of C9orf72, p62, α‐synuclein, and LC3 in preclinical PFF‐induced PD mice. (L) Statistical analysis of the relative content of C9orf72 in preclinical PFF‐induced PD mice. (M) Statistical analysis of the relative content of p62 in preclinical PFF‐induced PD mice. (N) Statistical analysis of the relative content of α‐synuclein in preclinical PFF‐induced PD mice. (O) Statistical analysis of the relative content of LC3, *n* = 3/group, **p < 0.01, *p < 0.05. Data are presented as the mean ± SEM.

To determine the involvement of C9orf72 in the pathogenesis of PD, we measured the expression of C9orf72 in 3 PD models with the environmental factor 1‐methyl‐4‐phenyl‐1,2,3,6 (MPTP), genetic factor α‐synuclein A53T, and preclinical factor recombinant α‐synuclein PFF. In MPTP‐treated PD mice, we noted decreased C9orf72 protein content in SNpc, dysregulated autophagy, and the accumulation of autophagic substrates (Figure 1A–E); moreover, the dopaminergic neuronal expression of tyrosine hydroxylase‐positive SNpc was decreased (Figure S1A,B). Then, we generated an adeno‐associated virus (AAV) of alpha‐synuclein A53T and an adeno‐associated virus (AAV) of recombinant alpha‐synuclein PFF. Accordingly, we found that the C9orf72 expression was downregulated in SNpc of PD mice treated with A53T adenovirus (Figure 1F,G), which was accompanied by dysregulated autophagy (Figure 1F,H–J), such as the downregulation of LC3II/I expression and the upregulation of p62 and α‐synuclein expression, and the DA neurons were lost in SNpc (Figure S1C,D). Treatment with recombinant PFF led to α‐synuclein phosphorylation to p‐α‐synuclein (Figure S1E) and the downregulation of the C9orf72 expression in the SNpc of mice (Figure 1K,L). The expression of dopaminergic neurons was decreased in tyrosine hydroxylase‐positive substantia nigra pars compacta (SNpc; Figure S1F,G), accompanied by dysregulated autophagy (Figure 1K,M–O).

### 3.2 C9orf72 protein is decreased in cellular models of PD

**FIGURE 2:**
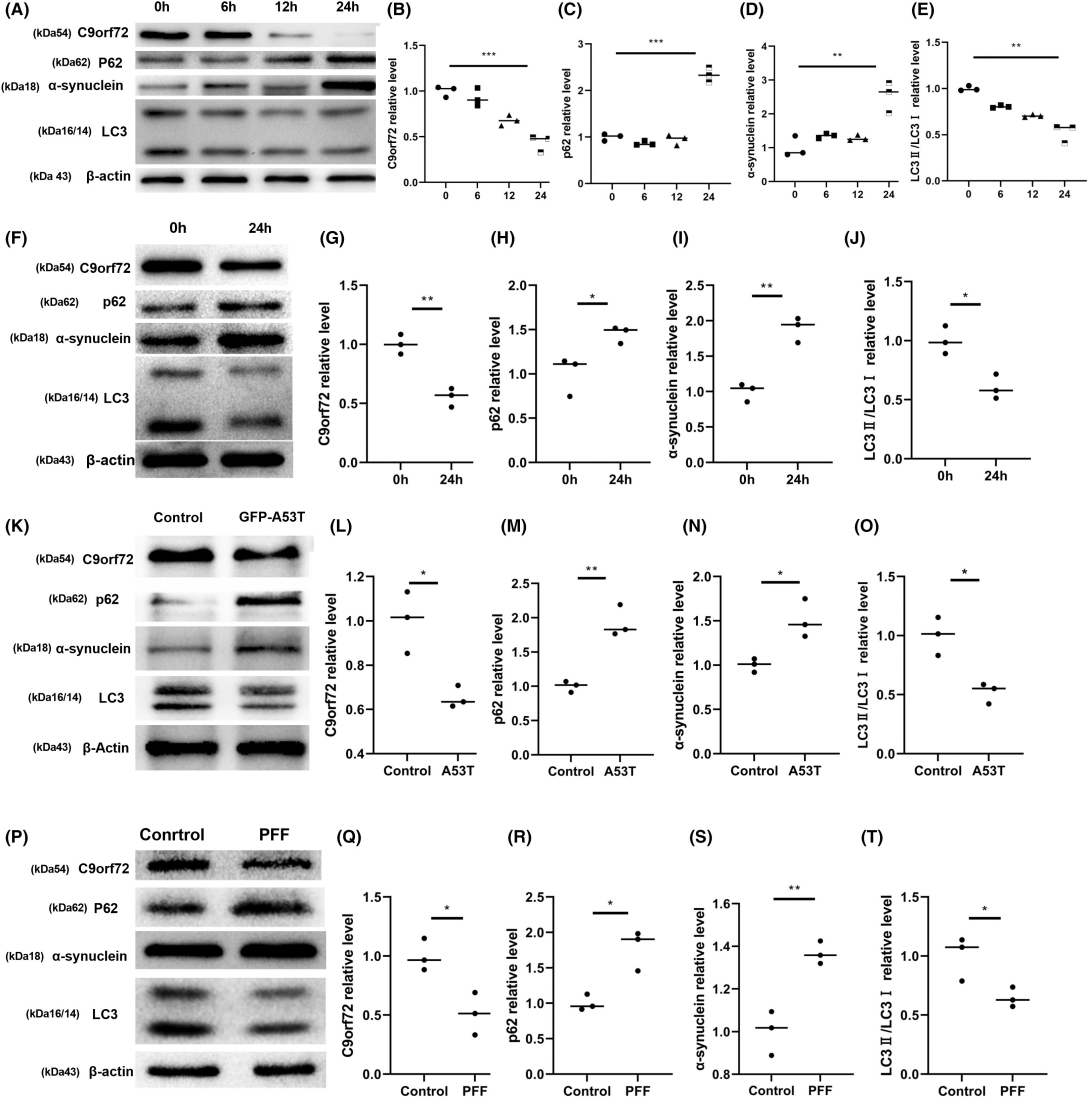
Chromosome 9 open reading frame 72 (C9orf72) expression was decreased in Parkinson's disease (PD) cellular models. (A) Western blotting of C9orf72, p62, α‐synuclein, and LC3 in MPP^+^‐induced neurons. (B) Statistical analysis of the relative content of C9orf72 in MPP^+^‐induced neurons. (C) Statistical analysis of the relative content of p62 in MPP^+^‐induced neurons. (D) Statistical analysis of the relative content of α‐synuclein in MPP^+^‐induced neurons. (E) Statistical analysis of the relative content of LC3 in MPP^+^‐induced neurons. (F) Western blotting of C9orf72, p62, α‐synuclein, and LC3 in MPP^+^‐induced SH‐SY5Y cells. (G) Statistical analysis of the relative content of C9orf72 in MPP^+^‐induced SH‐SY5Y cells. (H) Statistical analysis of the relative content of p62 in MPP^+^‐induced SH‐SY5Y cells. (I) Statistical analysis of the relative content of α‐synuclein in MPP^+^‐induced SH‐SY5Y cells. (J) Statistical analysis of the relative content of LC3 in MPP^+^‐induced SH‐SY5Y cells. (K) Western blotting of C9orf72, p62, α‐synuclein, and LC3 in an A53T‐induced SY5Y genetic model. (L) Statistical analysis of the relative content of C9orf72 in an A53T‐induced SY5Y genetic model. (M) Statistical analysis of the relative content of p62 in an A53T‐induced SY5Y genetic model. (N) Statistical analysis of the relative content of α‐synuclein in an A53T‐induced SY5Y genetic model. (O) Statistical analysis of the relative content of LC3 in an A53T‐induced SY5Y genetic model. (P) Western blotting of C9orf72, p62, α‐synuclein, and LC3 in a PFF‐induced neurons preclinical model. (Q) Statistical analysis of the relative content of C9orf72 in a PFF‐induced neurons preclinical model. (R) Statistical analysis of the relative content of p62 in a PFF‐induced neurons preclinical model. (S) Statistical analysis of the relative content of α‐synuclein in a PFF‐induced neurons preclinical model. (T) Statistical analysis of the relative content of LC3 in a PFF‐induced neurons preclinical model. *n* = 3/group, **p < 0.01, *p < 0.05. Data are presented as the mean ± SEM.

In addition to verifying the C9orf72 changes in PD models in vivo, we verified its expression changes in vitro. We also examined the expression of C9orf72 in 1‐methyl‐4‐phenyl‐pyridine ion (MPP^+^)‐exposed neurons and SY5Y cells, both of which indicated a decreased C9orf72 expression and the accumulation of autophagy substrates (Figure 2A–J). We then examined changes in the C9orf72 expression not only under the influence of the environmental factor MPP^+^ but also in the genetic model A53T as well as in the preclinical model PFF. The SY5Y cells and neurons exposed to α‐synuclein A53T demonstrated a decreased C9orf72 expression and dysfunctional autophagy, such as the downregulation of LC3II/I expression and the upregulation of p62 and α‐synuclein expression (Figure 2K–O; Figure S2F–J). Neurons and SY5Y treated with PFF showed the same changes, with reduced C9orf72 expression and dysregulated autophagy (Figure 2P–T, Figure S2K–O), whereas the messenger RNA (mRNA) levels were unchanged (Figure S2A–E). The results of animal and cellular experiments indicated that the regulation of C9orf72 downregulation was post‐translational.

### 3.3 Overexpression of C9orf72 activates autophagy and the knockdown of C9orf72 inhibits autophagy

**FIGURE 3:**
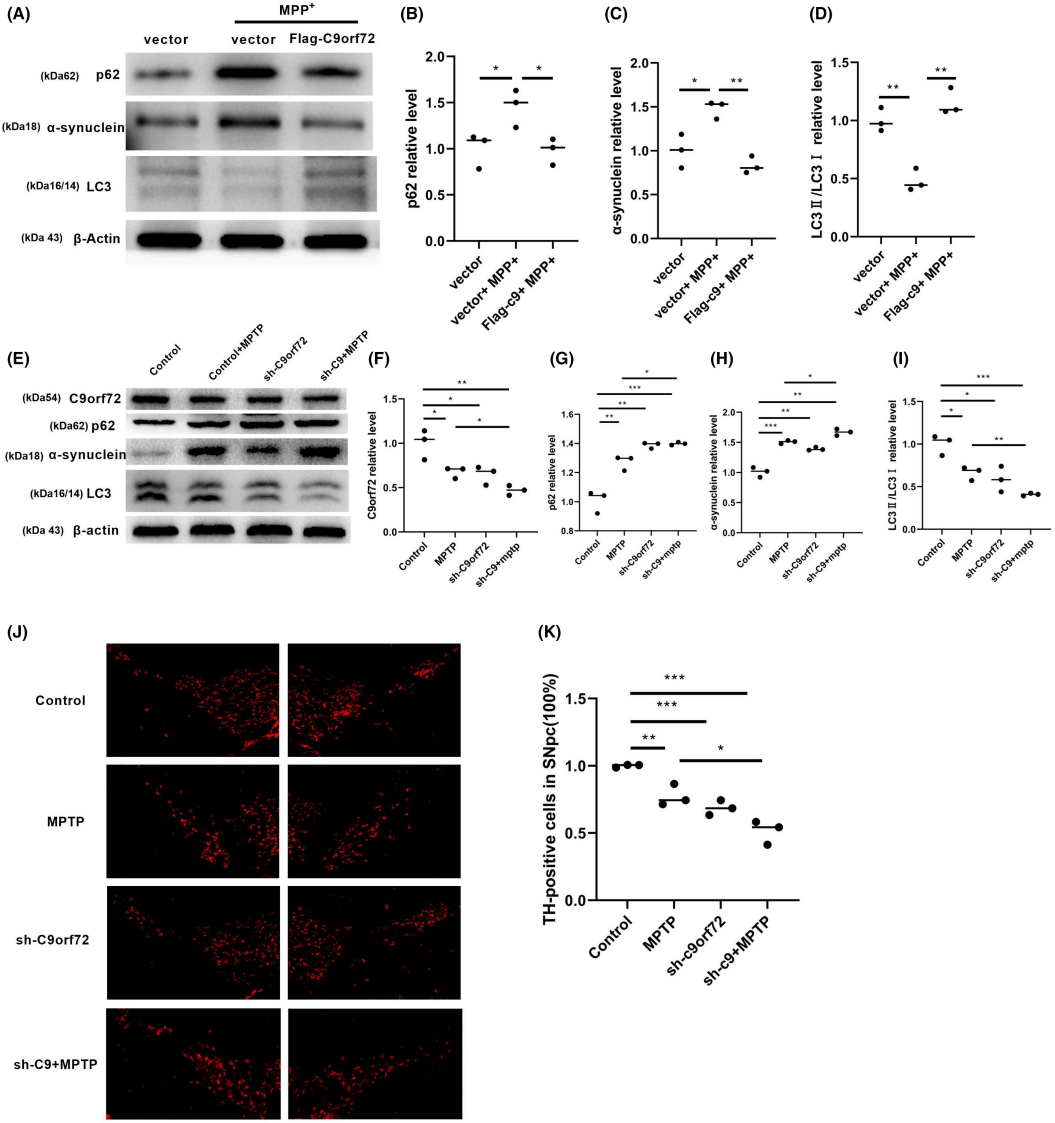
Knockdown of Chromosome 9 open reading frame 72 (C9orf72) aggravates autophagy and the overexpression of C9orf72 rescues autophagy. (A) Western blotting of p62, α‐synuclein, and LC3 in C9orf72‐overexpressing SY5Y cells induced by MPP^+^. (B) Statistical analysis of the relative content of p62 in C9orf72‐overexpressing SY5Y cells induced by MPP^+^. (C) Statistical analysis of the relative content of α‐synuclein in C9orf72‐overexpressing SY5Y cells induced by MPP^+^. (D) Western blotting of LC3 in C9orf72‐overexpressing SY5Y cells induced by MPP^+^. (E) Western blotting of C9orf72, p62, α‐synuclein, and LC3 in C9orf72‐knockdown mice. (F) Statistical analysis of the relative content of C9orf72 in C9orf72‐knockdown mice. (G) Statistical analysis of the relative content of p62 in C9orf72‐knockdown mice. (H) Western blotting of α‐synuclein in C9orf72‐knockdown mice. (I) Statistical analysis of the relative content of LC3 in C9orf72‐knockdown mice. *n* = 3/group, ***p < 0.001, **p < 0.01, *p < 0.05. (J) Immunofluorescence staining of TH enzyme in C9orf72‐knockdown mice. (K) TH+ neuron count and statistical analysis in C9orf72‐knockdown mice. *n* = 3/group. ***p < 0.001, **p < 0.01, *p < 0.05. Data are presented as the mean ± SEM.

In our past experiments, we demonstrated that C9orf72 was downregulated in an MPTP‐induced mouse model and cellular model, which was induced by MPP^+^ and PFF. However, the specific relationship between the expression of C9orf72 and PD remains unknown. Accordingly, we generated a Flag‐C9orf72 plasmid that overexpressed C9orf72 in SY5Y cells. To verify that the overexpression of C9orf72 can activate autophagy and rescue cell death, we performed cell viability tests (Figure S3A) and found that the C9orf72 overexpression protected SY5Y cells from MPP^+^ or PFF‐induced cell death, which signified that the overexpression of C9orf72 rescues autophagy impairment (Figure 3A–D, Figures S3B–E). Moreover, we constructed an adeno‐associated virus sh‐C9orf72 (AAV) that downregulated the C9orf72 protein expression. Our results revealed that the knockdown of C9orf72 expression led to aggravated MPTP‐induced autophagy dysfunction (Figure 3E–I), while the (TH+) dopaminergic neuron expression was decreased in SNpc (Figure 3J,K).

### 3.4 C9orf72 is phosphorylated and degraded by the ubiquitin‐proteasome pathway

**FIGURE 4:**
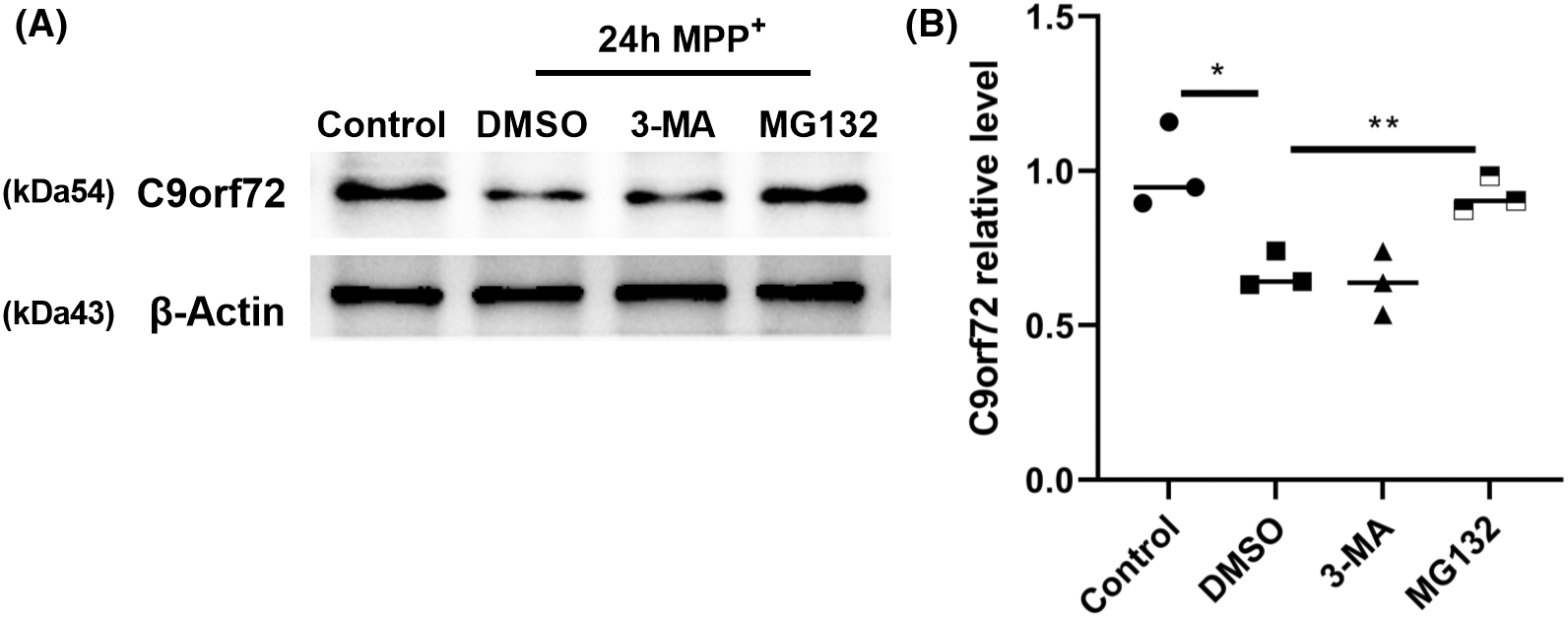
Chromosome 9 open reading frame 72 (C9orf72) protein is degraded by the ubiquitination degradation pathway. (A) 3‐MA and MG132 were used to intervene in the autophagy‐lysosomal pathway and the ubiquitin‐proteasome pathway, respectively, in order to detect the degradation of C9orf72 protein after MPP^+^ treatment of primary neurons for 24 h. (B) Statistical analysis of the relative content of C9orf72. *n* = 3, *p < 0.05. Data are presented as the mean ± SEM.

Our past results suggested that, in the PD model, the mRNA expression of C9orf72 did not change; rather, the protein expression decreased, which implies no issues with the transcription process of C9orf72 in PD, although protein degradation did occur. This observation raised the question regarding the mechanism of C9orf72 protein degradation. There are two common protein‐degradation pathways: the autophagy‐lysosomal degradation pathway and the ubiquitin‐proteasome degradation pathway. In order to clarify the degradation pathway of C9orf72 protein in PD, we evaluated the degradation pathway of C9orf72 protein by adding autophagy‐lysosomal pathway inhibitor 3‐MA and ubiquitinated proteasome pathway inhibitor MG132 to neuronal cells. The addition of MG132 was found to inhibit the degradation of C9orf72 protein induced by MPP^+^ or A53T, whereas the addition of 3‐MA had a negligible effect, indicating that C9orf72 was not degraded by the autophagoproteasome pathway, but rather by the ubiquitin‐proteasome pathway (Figure 4A,B; Figure S4A,B).

### 3.5 Cdk5 can mediate its degradation by phosphorylating C9orf72

**FIGURE 5:**
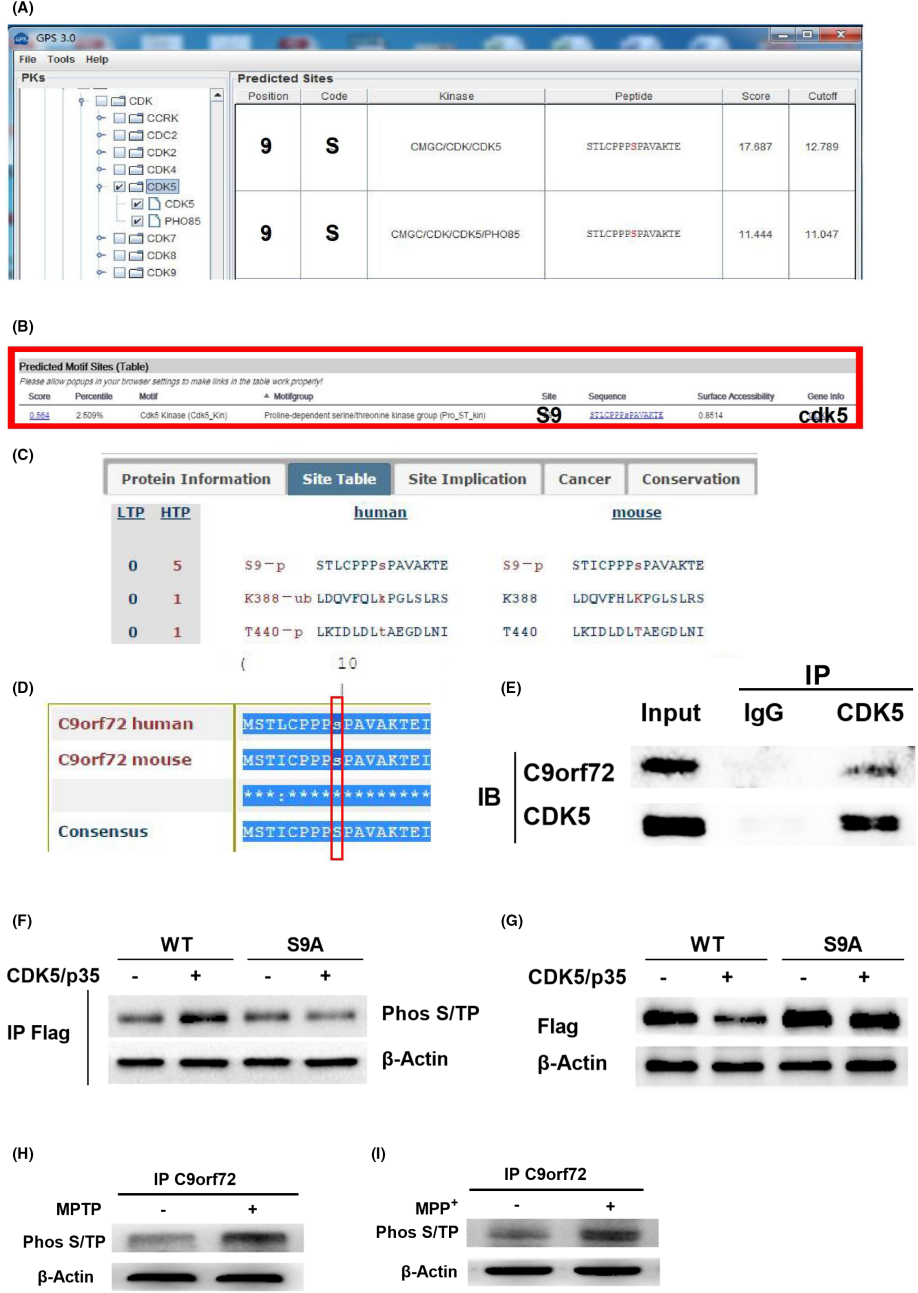
Cdk5 is involved in the degradation of Chromosome 9 open reading frame 72 (C9orf72). (A) GPS3.0 predicts that the S9 site of the C9orf72 protein can be phosphorylated by Cdk5. (B) Scansite predicted that Cdk5 phosphorylates the S9 site of the C9orf72 protein. (C) PhosphoSitePlus revealed that the cC9orf72 S9 site could be phosphorylated in the mass spectrometry results of other scholars. (D) DNAMAN alignment of the known amino acid conservation at the S9‐site vicinity of human and mouse C9orf72 proteins. (E) C9orf72 interacted with Cdk5. (F) HEK293 cells were co‐transfected with HA‐Cdk5/Myc‐p35, Flag‐C9orf72 WT plasmid, or Flag‐C9orf72 S9A plasmid, and Phos S/TP antibody was used to detect the phosphorylation results. (G) After 24 h of HEK293 cells' transfection with HA‐Cdk5/Myc‐p35, wild‐type Flag‐C9orf72 WT, or mutant Flag‐C9orf72 S9A plasmid. Western blotting to detect the expression of C9orf72 protein. (H) Detection of the phosphorylation level of C9orf72 protein in MPTP‐induced mice. (I) Detection of the phosphorylation level of C9orf72 protein in MPP^+^‐induced primary neurons.

A search using the bioinformatics software PhosphoSite PLUS revealed that the S9 site of the C9orf72 protein could be phosphorylated and that the combination of GPS 3.0, SCANSITE, PhosphoSite PLUS, and other sequence‐based phosphorylation site‐prediction software and sequence analysis software were used to phosphorylate the C9orf72 protein. We analyzes the possible sites of phosphorylation of C9orf72 by CDK5 kinase, which further revealed that Cdk5 was the most likely protein kinase to phosphorylate the C9orf72 protein (Figure 5A–D). To confirm the role of Cdk5 in C9orf72 degradation, we conducted co‐immunoprecipitation experiments in neurons (Figure 5E). We accordingly designed a Flag‐C9orf72 plasmid (WT) and a mutant of Flag‐C9orf72, wherein Ser9 was mutated to alanine (Ser9A). WT or S9A C9orf72 was then co‐transfected with HA‐Cdk5/Myc‐p35 into HEK293 cells. The Cdk5/p35 complex phosphorylated Flag‐C9orf72 (WT), but not Ser9A mutant in vitro. Ser9A mutants exhibited lower phosphorylation levels relative to WT (Figure 5F; Figure S5A). The Cdk5/p35 complex promoted the degradation of C9orf72 protein after the phosphorylation of Flag‐C9orf72 (WT) in vitro, while the Ser9A mutant C9orf72 protein did not degrade as it could not be phosphorylated (Figure 5G, Figure S5B). In addition, the phosphorylation level of the C9orf72 protein was found to have increased in PD cells and the animal models (Figure 5H,I).

### 3.6 Cdk5 inhibitor reverses autophagy impairment in PD

**FIGURE 6:**
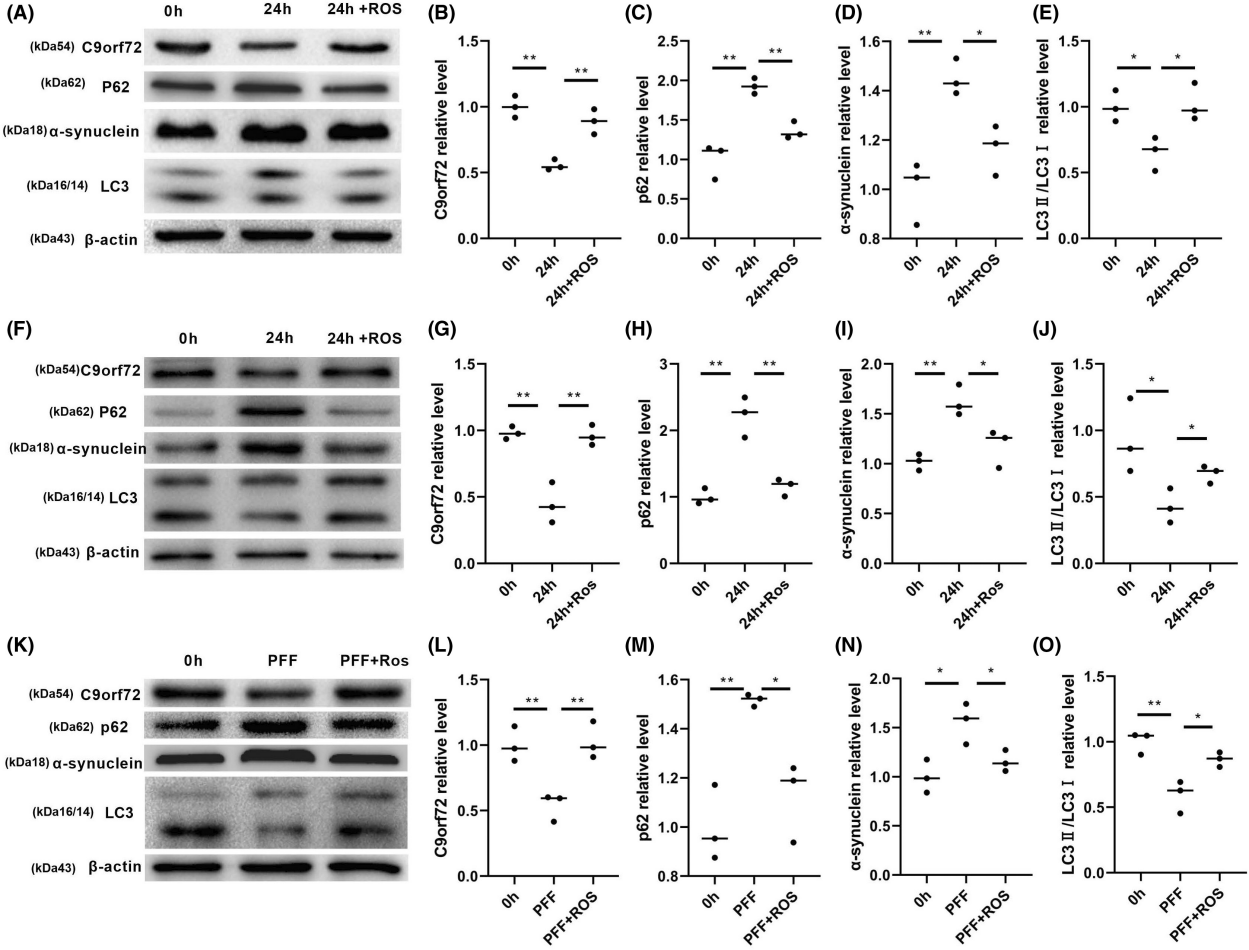
Cdk5 inhibitors inhibited the phosphorylation degradation of Chromosome 9 open reading frame 72 (C9orf72). (A) Western blotting of Cdk5 inhibitor ROSCOVITINE on the expression of C9orf72, p62, α‐synuclein, and LC3 in MPP^+^‐induced neurons. (B) Statistical analysis of the relative content of C9orf72 in MPP^+^‐induced neurons inhibited by ROSCOVITINE. (C) Statistical analysis of the relative content of p62 in MPP^+^‐induced neurons inhibited by ROSCOVITINE. (D) Statistical analysis of the relative content of α‐synuclein in MPP^+^‐induced neurons inhibited by ROSCOVITINE. (E) Statistical analysis of the relative content of LC3 in MPP^+^‐induced neurons inhibited by ROSCOVITINE. (F) Western blotting of the Cdk5 inhibitor ROSCOVITINE on the expression of C9orf72, p62, α‐synuclein, and LC3 in MPP^+^‐induced SY5Y cells. (G) Statistical analysis of the relative content of C9orf72 in MPP^+^‐induced SY5Y cells inhibited by ROSCOVITINE. (H) Statistical analysis of the relative content of p62 in MPP^+^‐induced SY5Y cells inhibited by ROSCOVITINE. (I) Statistical analysis of the relative content of α‐synuclein in MPP^+^‐induced SY5Y cells inhibited by ROSCOVITINE. (J) Statistical analysis of the relative content of LC3 in MPP^+^‐induced SY5Y cells inhibited by ROSCOVITINE. (K) Western blotting of the Cdk5 inhibitor ROSCOVITINE on the expression of C9orf72, p62, α‐synuclein, and LC3 in PFF‐induced neurons. (L) Statistical analysis of the relative content of C9orf72 in PFF‐induced neurons inhibited by ROSCOVITINE. (M) Statistical analysis of the relative content of p62 in PFF‐induced neurons inhibited by ROSCOVITINE. (N) Statistical analysis of the relative content of α‐synuclein in PFF‐induced neurons inhibited by ROSCOVITINE. (O) Statistical analysis of the relative content of LC3 in PFF‐induced neurons inhibited by ROSCOVITINE. *n* = 3/group, **p < 0.01, *p < 0.05. Data are presented as the mean ± SEM.

We demonstrated, in our past experiments, that C9orf72 was downregulated in MPP + ‐induced cell models of PD. C9orf72 was degraded by the phosphorylation of Cdk5. We, therefore, used the Cdk5 inhibitor ROSCOVITINE to inhibit the phosphorylation of C9orf72 by Cdk5^26^ and tested whether the degradation of C9orf72 could be rescued by ROSCOVITINE. Our experimental results demonstrated that ROSCOVITINE improved the MPP+ degradation of C9orf72 in neurons and SY5Y cells (Figure 6A–J) and that ROSCOVITINE ameliorated the degradation of C9orf72 by PFF in the neurons (Figure 6K–O).

### 3.7 Myr‐C9orf72 interference peptide rescues neuronal loss and motor dysfunction

**FIGURE 7:**
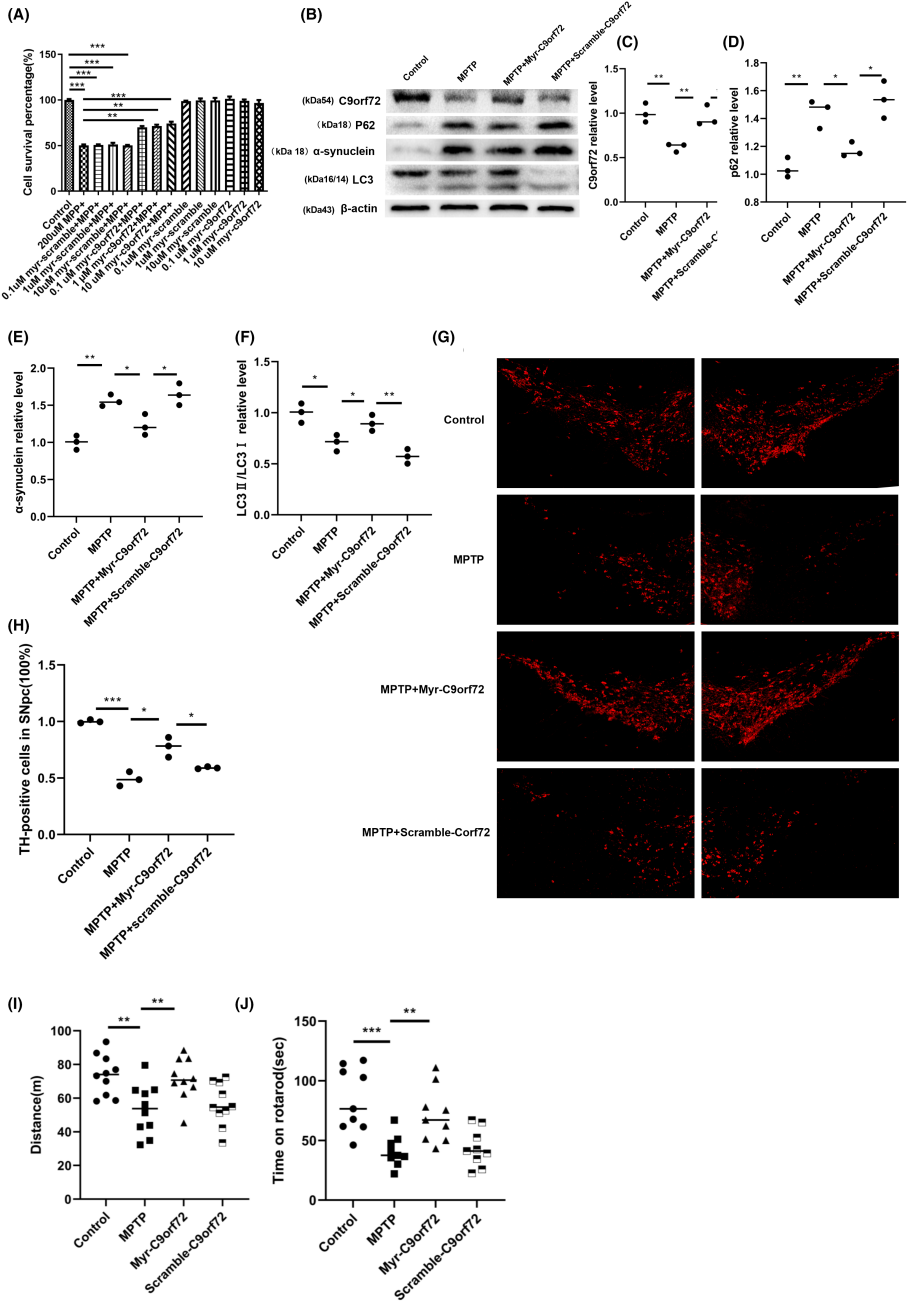
Myr‐Chromosome 9 open reading frame 72 (C9orf72) peptide rescues neuronal loss and motor dysfunction. A The effect of different concentrations of Myr‐C9orf72 peptide on the viability of MPP^+^‐treated primary neurons. *n* = 3. ***p < 0.001, **p < 0.01. B Western blotting of C9orf72, p62, α‐synuclein, and LC3. C Statistical analysis of the relative content of C9orf72. D Statistical analysis of the relative content of p62. E Western blotting of α‐synuclein. F Statistical analysis of the relative content of LC3. *n* = 3/group, **p < 0.01, *p < 0.05. G Immunofluorescence staining of TH. H TH+ neuron count and statistical analysis. *n* = 3/group. ***p < 0.001, *p < 0.05. I Rotarod test data after the experiment. J Open field data after the experiment. *n* = 12/group, compared with the control group, ***p<0.001, **p<0.01. Data are presented as the mean ± SEM.

**FIGURE 8:**
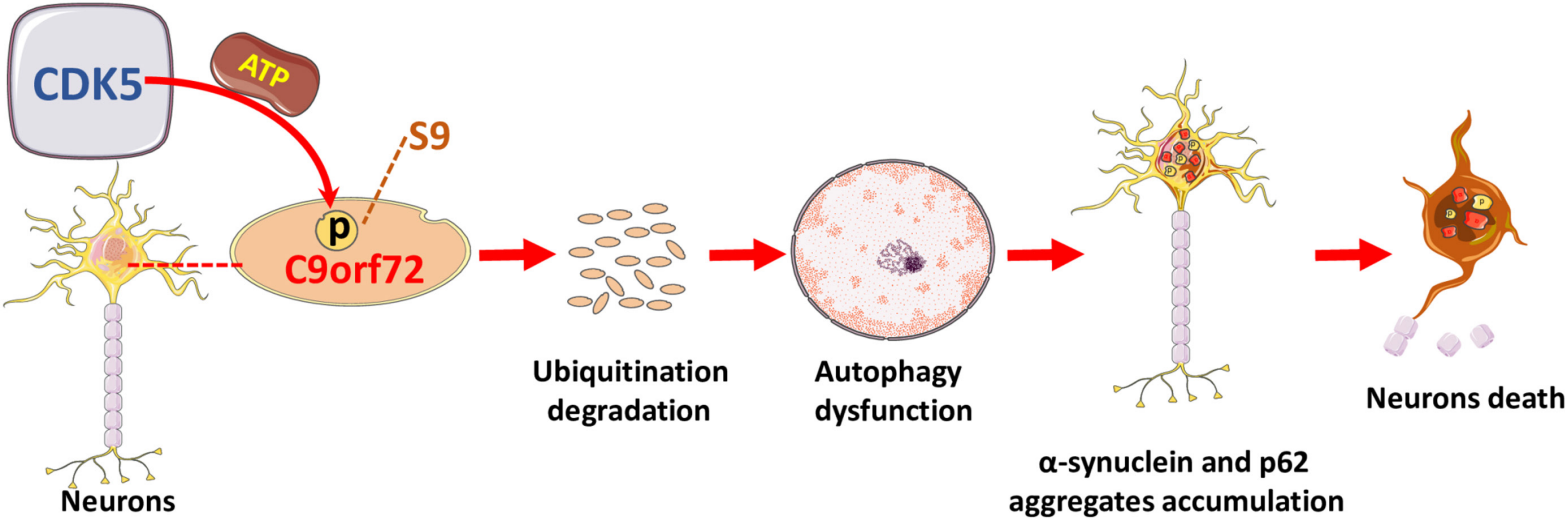
The theoretical scheme of Chromosome 9 open reading frame 72 (C9orf72) protein involved in neuronal death in Parkinson's disease (PD). During PD, Cdk5 is activated in dopaminergic neurons of the substantia nigra, and the activated protein kinase Cdk5 phosphorylates the S9 site of the C9orf72 protein. Phosphorylation at the S9 site mediates C9orf72 protein degradation through the ubiquitin‐proteasome pathway. The degradation and loss of function of the C9orf72 protein led to autophagy impairment in neurons. Proteins such as p62 and α‐synuclein, which cannot be effectively cleared by autophagy, accumulate abnormally, and their neurotoxicity causes the death of dopaminergic neurons.

Based on our cumulative findings, we designed a short peptide named Myr‐C9orf72 peptide (LCPPPSPAVAK, 4–14), which competitively inhibited Cdk5‐dependent Ser9 phosphorylation in C9orf72. This peptide was conjugated to myristic acid to ensure its cell‐penetrating ability. Unsurprisingly, the Myr‐C9orf72 peptide effectively protected MPP^+^‐induced neuronal death (Figure 7A). Considering the protective efficacy of the Myr‐C9orf72 peptide in vitro, we assessed its effect on SNpc DA neuron loss and motor function performance in MPTP‐treated WT mice. Consistent with these findings in MPP^+^‐treated primary cortical neurons, Myr‐C9orf72 peptide inhibited the degradation of C9orf72 caused by MPTP (Figure 7B,C), alleviated the abnormal autophagy function (Figure 7B,D–F), and the death of dopaminergic neurons in PD, while effectively protecting the neurons (Figure 7G,H), and the damage of motor function can be alleviated(Figure 7I–L). Overall, during PD, Cdk5 was activated in dopaminergic neurons of the substantia nigra, and the activated protein kinase Cdk5 phosphorylated the S9 site of the C9orf72 protein. Phosphorylation at the S9 site mediated C9orf72 protein degradation through the ubiquitin‐proteasome pathway. The degradation and loss of function of the C9orf72 protein led to autophagy impairment in neurons. Proteins such as p62 and α‐synuclein that could not be effectively cleared by autophagy accumulated abnormally, and their neurotoxicity caused the death of dopaminergic neurons (Figure 8). Our results exhibited that competitive inhibition by Cdk5‐dependent phosphorylation of C9orf72 attenuated neuronal death in an MPP^+^‐treated cellular model and an MPTP‐treated mouse model of PD.

### 3.8 Statistical analysis

Software SPSS 22.0 and GraphPad Prism 9.0 were used for data analysis, and the data were expressed as the mean ± standard error of the mean (SEM). For pairwise comparisons, an unpaired *t*‐test was applied. For comparisons of more than two treatment groups, analysis of variance (ANOVA) and Tukey's posthoc test were performed. *p* < 0.05 was considered to indicate a statistically significant difference (*p < 0.05, **p < 0.01, ***p < 0.001, and ****p < 0.0001).

## 4 DISCUSSION

In recent years, the role of C9orf72 in neurodegenerative diseases has received increasing attention, with reports of its expression changes in AD and ALS,^5^, ^6^, ^7^ albeit the specific underlying mechanism remains unclear. Presently, there is no literature on the relationship between C9orf72 and PD. The C9orf72 protein can induce diseases by affecting the transcription of genes,^27^ such as interfering with the translation of RNA into protein and the transportation of RNA within cells. Pathogenicity can also be induced by genetic mutations that form toxic dipeptide repeat protein (DPR) aggregates through repeat‐associated non‐AUG translation and sense or antisense extended RNAs.^28^, ^29^, ^30^, ^31^ We found that the C9orf72/SMCR8/WDR41 complex was important for the recruitment of autophagy‐initiation complexes FIP200/ULK‐1/ATG13/ATG10 and RAB8a/RAB39b to form autophagosomes and that C9orf72 could regulate autophagy initiation by regulating the phosphorylation at Ser 757 of ULK1.^32^ Therefore, we believe that the association between the autophagy disorder caused by the loss of C9orf72 protein function and the pathogenesis of PD is worth exploring.

We applied the neurotoxic drug MPTP to simulate the environmental factors, α‐synuclein A53T adenovirus injection to simulate genetic factors, and α‐synuclein PFF adenovirus injection to simulate the preclinical factors to induce PD. We recorded behavioral changes in mice in the animal models (except in the preclinical models); the dopaminergic neurons were lost, and the C9orf72 expression was significantly reduced, accompanied by autophagy dysfunction and the accumulation of autophagy substrates. The corresponding results were confirmed at the cellular level. However, the expression of C9orf72 mRNA did not decrease, indicating that the expression change of C9orf72 in PD was degraded at the protein level.

In addition, to further verify the relationship between C9orf72 and autophagy, we constructed sh‐C9orf72 adenovirus to downregulate the expression of C9orf72 protein and evaluated whether the reduction of the C9orf72 protein level could lead to autophagy disorder. The experimental results revealed that the downregulation of C9orf72 protein expression led to autophagy dysfunction, such as the downregulation of LC3II/I expression and the upregulation of p62 and α‐synuclein expression. We also constructed the Flag‐C9orf72 plasmid to construct a model for overexpressing C9orf72 in SH‐SY5Y cells and neurons. The model proved that, after the overexpression of C9orf72, the expression levels of p62 and α‐synuclein were reduced when compared with those in the vector group, thereby demonstrating that the overexpression of C9orf72 activated autophagy and promoted the degradation of autophagy substrates, upregulated the expression of LC3II/I, and downregulated the expression of p62 and α‐synuclein. However, whether the overexpression of C9orf72 led to the same autophagy‐activating effect in animal models warrants further in‐depth studies.

Cdk5, a special member of the cyclin‐dependent protein kinase (Cdk) family, is considered a neuron‐specific kinase that plays an important role in several cellular functions, including cell motility and survival. It exerts its biological effects by phosphorylating serine/threonine.^33^ Past studies have revealed that Cdk5 is overactivated in the brain during PD and that its phosphorylation function is enhanced.^22^, ^34^ Accordingly, we speculated that Cdk5 may be involved in the degradation of C9orf72. Dysregulated Cdk5 was found to be associated with neuronal death by the Cdk5 substrate endophilin B1(EndoB1). In addition, Cdk‐mediated phosphorylation of EndoB1 ensured autophagy‐induced neuronal loss in a PD model.^35^, ^36^ Our experiments confirmed that C9orf72 was degraded through the ubiquitin‐proteasome pathway and that the degradation of C9orf72 was dependent on the phosphorylation of Cdk5. In this way, we reversed the degradation level of C9orf72 by inhibiting the phosphorylation level of Cdk5 in order to achieve the purpose of intervention in the pathogenesis of PD.

Furthermore, we tested whether the decrease in C9orf72 expression could be slowed down by inhibiting the Cdk5 activity with the Cdk5 inhibitor ROSCOVITINE. Our experimental results revealed that, in SH‐SY5Y and primary neuron cell models, the protein expression of C9orf72 in the ROSCOVITINE group was higher than that in the MPP+ group, implying that Cdk5 was indeed involved in the degradation process of C9orf72. However, Cdk5 possessed several substrates in the nervous system and directly inhibited the activity of Cdk5 kinase, which led to other dysfunctions of the body in vivo. To specifically interfere with Cdk5 on the S9 position of the C9orf72 protein, a polypeptide containing the Ser9 site of the C9orf72 protein, amino acids 4‐14, sequence A: LCPPPSPAVAK was designed. In order to improve its ability to penetrate the cell membrane, myristic acid (Myr) was combined at the N‐terminus of the short peptide. The Myr‐C9orf72 4‐14 peptide targeted and competed with endogenous C9orf72 for binding to Cdk5 in the cells, thereby specifically interfering with Cdk5 phosphorylation of the Ser9 site of the C9orf72 protein. Our experimental results indicated that the Myr‐C9orf72 peptide could effectively protect the neurons in the PD model. Myr‐C9orf72 transmembrane peptide attenuates abnormal autophagy, such as the downregulation of LC3II/I expression, upregulation of p62 and α‐synuclein expression, and dopaminergic neuron death and dyskinesia in PD by interfering with Cdk5 phosphorylation of the Ser9 site of C9orf72 protein. Moreover, Myr‐C9orf72 peptide inhibited the effect of decreased C9orf72 expression in an MPTP‐induced mouse PD model.

Presently, the progress of pharmacological intervention in PD is limited to symptomatic treatment, which raises the question of whether effective neuroprotective drugs can be developed to treat symptoms along with delaying the disease progression caused by neuronal death. Our study results provide new ideas and directions for elucidating the mechanism and drug treatment of dopaminergic neuron death during the onset of PD, indicating that C9orf72 provides strong evidence supporting the potential of C9orf72 as a PD therapeutic target. Further research can clinically verify the efficacy and safety of the peptide drug Myr‐C9orf72.

In summary, our cumulative results demonstrated that, in PD, the expression level of the C9orf72 protein was significantly reduced and that the key nervous system kinase Cdk5 directly phosphorylated the S9 site of the C9orf72 protein. Specifically, interfering with Cdk5 phosphorylation of the S9 site of C9orf72 alleviated the autophagy dysfunction, such as the downregulation of LC3II/I expression and the upregulation of p62 and α‐synuclein expression, which also alleviated the neuronal death and motor dysfunction mediated by C9orf7 protein degradation.

## AUTHOR CONTRIBUTIONS

All authors participated in the design and execution of the experiments. Xingfeng Xu and Jianguo Yan wrote this article. Data were analyzed by Yan Su, Mao Li, and Qi Wang, who also reviewed the manuscript. Jianguo Yan and Yali Zhou directed the research and revised the article.

## FUNDING INFORMATION

The author(s) disclosed receipt of the following financial support for the research, authorship, and/or publication of this article. This work was supported by the Natural Science Foundation of Guangxi Province (No. 2023GXNSFAA026213), the National Natural Science Foundation of China [NSFC, No. 81860246, No. 82160517], Innovation Project of Guangxi Graduate Education (No. YCSW2023417), the Scientific Research and Technology Development Program of Guangxi [grant number AD18281009], Thousands of Young and Middle‐aged Backbone Teachers in Guangxi colleges and Universities Training Plan, and Guangxi Medical and health key cultivation discipline construction project.

## CONFLICT OF INTEREST STATEMENT

The authors have declared that no competing interest exists.

## CONSENT FOR PUBLICATION

All authors have consented to the submission of the manuscript.

## CONSENT TO PARTICIPATE

Not applicable.

## Supporting information


Fig. S1.

Fig. S2.

Fig. S3.

Fig. S4.

Fig. S5.

## Data Availability

The data supporting the present findings are available within the article and Supplementary Information files. The data for this study are available from the corresponding author upon reasonable request.
