# Supplementary figures and images for: Cdk5 phosphorylation‐dependent C9orf72 degradation promotes neuronal death in Parkinson's disease models
Source: CNS Neurosci Ther. 2023 Jun 23;29(12):3952–66. doi: 10.1111/cns.14319 (PMC10651984; doi:10.1111/cns.14319)

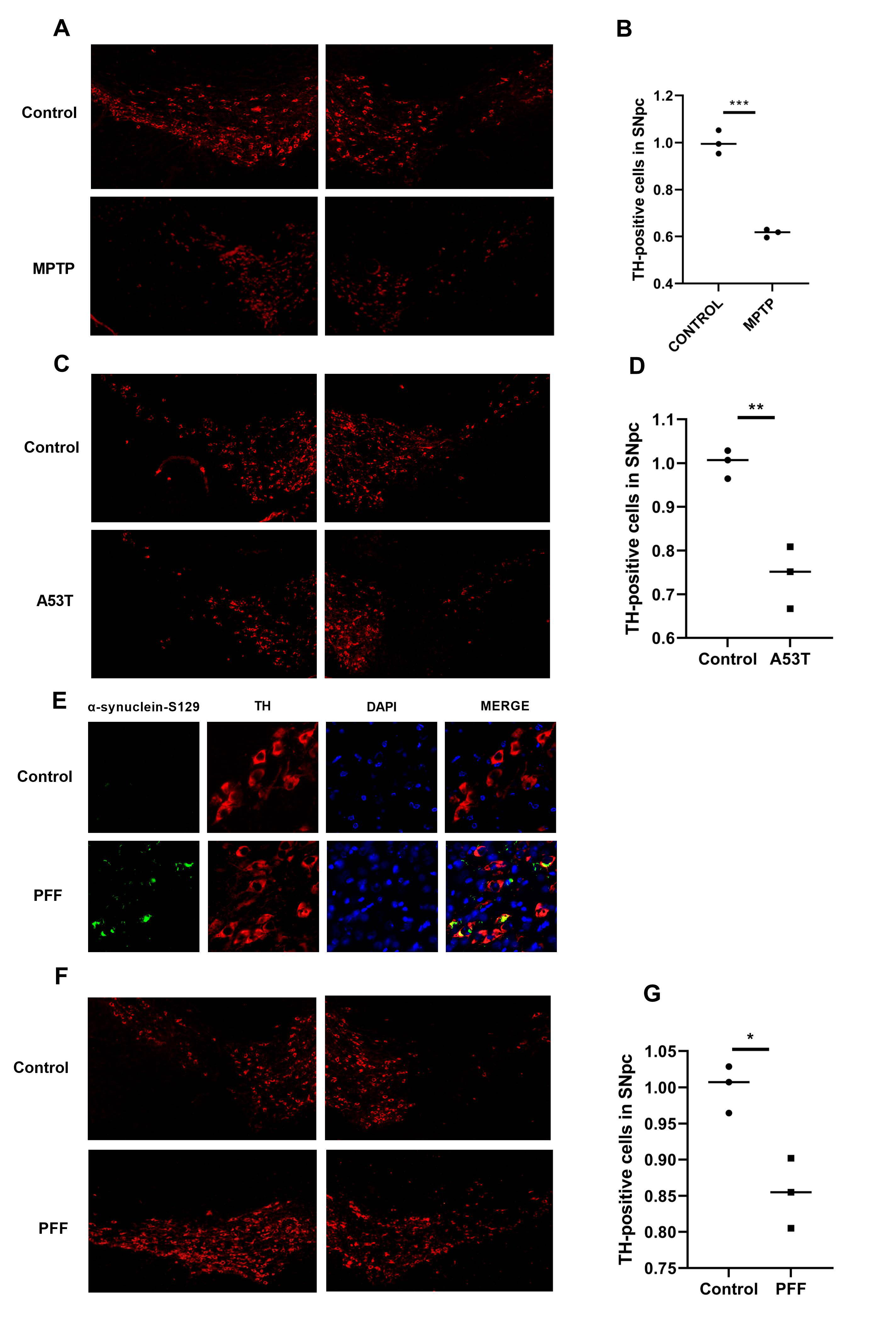

Supplement: Supplementary file 1 — Fig. S1. [file CNS-29-3952-s004.tif]

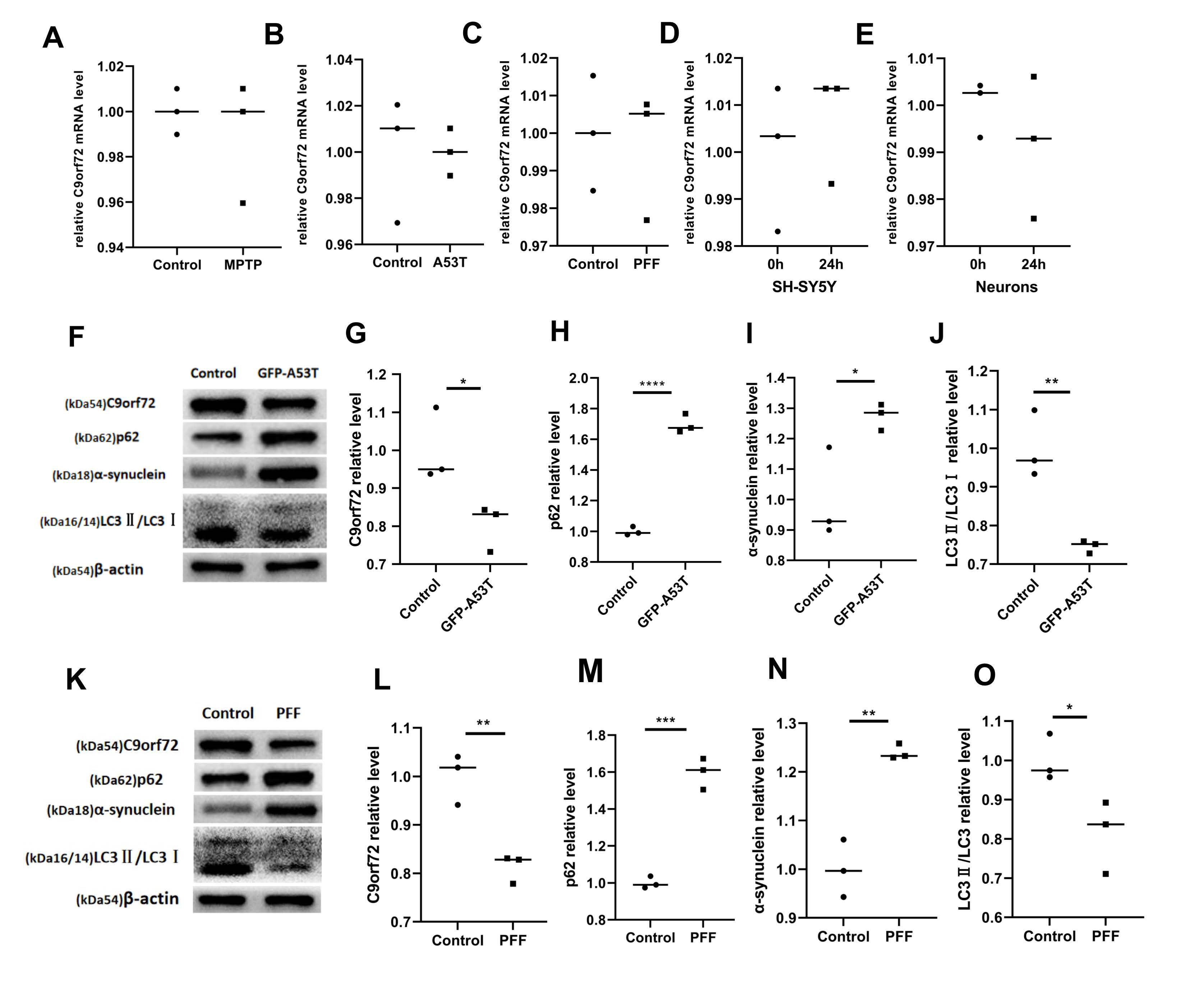

Supplement: Supplementary file 2 — Fig. S2. [file CNS-29-3952-s005.tif]

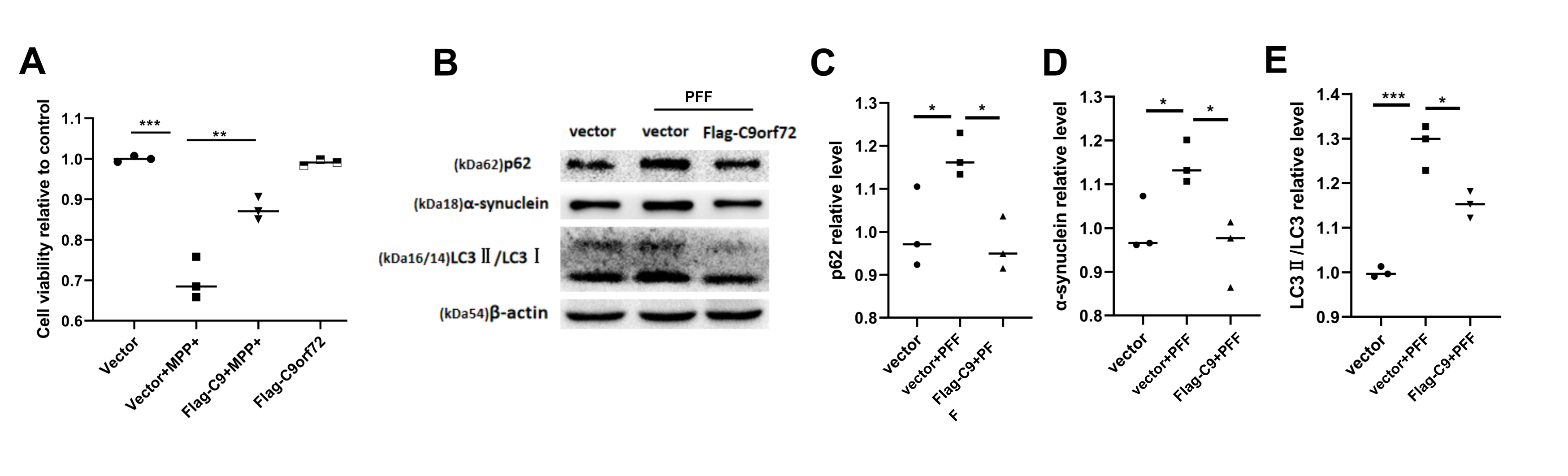

Supplement: Supplementary file 3 — Fig. S3. [file CNS-29-3952-s001.tif]

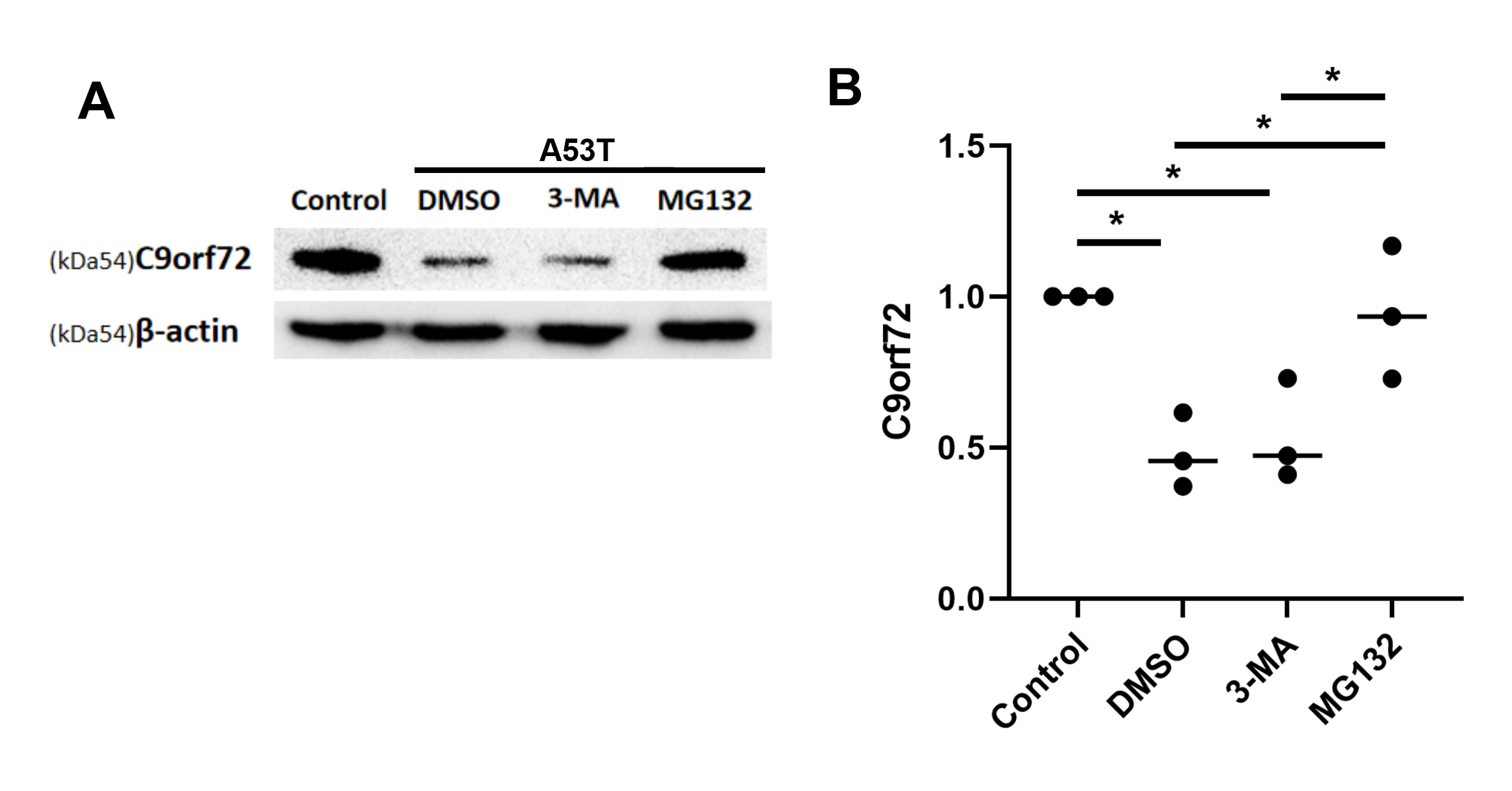

Supplement: Supplementary file 4 — Fig. S4. [file CNS-29-3952-s003.tif]

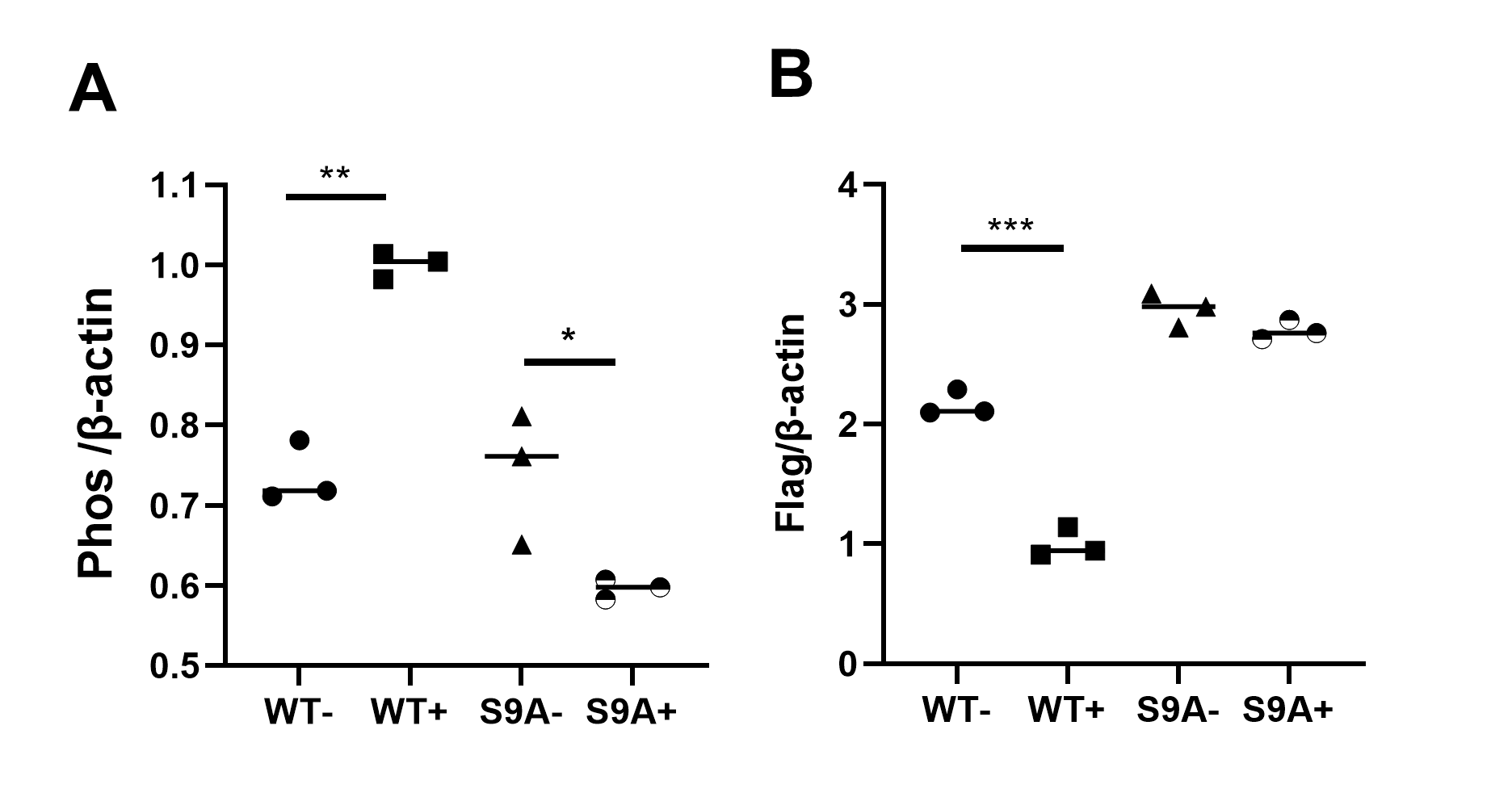

Supplement: Supplementary file 5 — Fig. S5. [file CNS-29-3952-s002.tif]
